# Digital Imaging in Cytopathology

**DOI:** 10.4061/2011/264683

**Published:** 2011-07-19

**Authors:** Walid E. Khalbuss, Liron Pantanowitz, Anil V. Parwani

**Affiliations:** ^1^Division of Pathology Informatics, Department of Pathology, University of Pittsburgh Medical Center, Pittsburgh, PA 15232, USA; ^2^Division of Cytology, UPMC Shadyside Hospital, 5150 Centre Avenue, POB2, Suite 201, Pittsburgh, PA 15232, USA

## Abstract

Rapid advances are occurring in the field of cytopathology, particularly in the field of digital imaging. Today, digital images are used in a variety of settings including education (E-education), as a substitute to multiheaded sessions, multisite conferences, publications, cytopathology web pages, cytology proficiency testing, telecytology, consultation through telecytology, and automated screening of Pap test slides. The accessibility provided by digital imaging in cytopathology can improve the quality and efficiency of cytopathology services, primarily by getting the expert cytopathologist to remotely look at the slide. This improved accessibility saves time and alleviates the need to ship slides, wait for glass slides, or transport pathologists. Whole slide imaging (WSI) is a digital imaging modality that uses computerized technology to scan and convert pathology and cytology glass slides into digital images (digital slides) that can be viewed remotely on a workstation using viewing software. In spite of the many advances, challenges remain such as the expensive initial set-up costs, workflow interruption, length of time to scan whole slides, large storage size for WSI, bandwidth restrictions, undefined legal implications, professional reluctance, and lack of standardization in the imaging process.

## 1. Introduction

Digital images are increasingly being used in the field of cytopathology for tele-education, clinical consultation, telecytology, remote conferences, web-based learning, quality assurance, and secondary applications such as image analysis [1–12]. 

**Figure 1:**
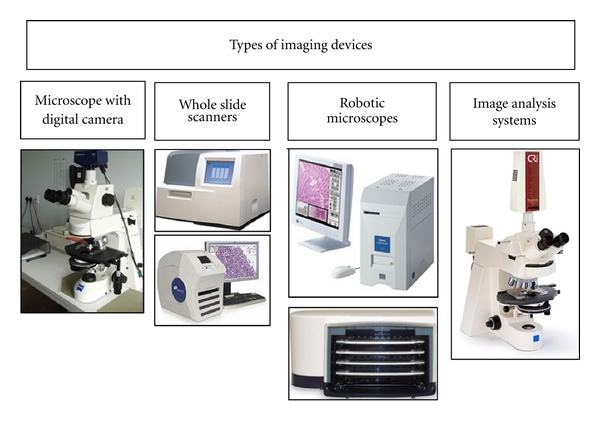
Input devices for creating digital images: (far left) digital camera attached via c-mount adapter to a Zeiss light microscope, (middle left) whole slide scanners showing (upper) the Omnyx VL4 whole slide scanner that scans up to 4 slides at a time and (lower) the Aperio Scanscope CS Scanner, (middle right) robotic microscopes including (upper) the Nikon CoolScope II, one glass slide scanner and (lower) the Trestle 5L50, 50 slide loaders (far right) Cambridge Research and Instrumentation (CRi) Nuance multispectral imaging (MSI) Camera.

A digital image is represented in a computer by a two-dimensional array of numbers, each element of which represents a pixel (*pic*ture *e*lement). Digital images can be created by a variety of input devices such as a digital camera. The imaging process involves capturing, saving (storage), editing (if necessary), and sharing (viewing, displaying, printing) digital images [1]. Cytopathology has unique imaging needs such as liquid-based cytology which offers an advantage of uniformly fixed and stained cellular areas that are relatively small to be imaged with high-quality viewing [13]. There are multiple types of images that can be used to acquire digital images (Figure 1). Microscopic digital images can be static (still images), viewed live (real-time robotic microscopy), or viewed after scanning of the glass slides (whole slide digital imaging (WSI) or virtual microscopy) [1, 3, 8]. Efforts are underway to standardize the process of acquiring, storing, and displaying digital images in pathology similar to radiology [14–16]. 

In the field of cytopathology, digital images are used in a variety of settings including education (E-education), as a substitute to multiheaded sessions, multisite conferences, publications, cytopathology web pages, cytology proficiency testing, telecytology, consultation through telecytology, and automated screening of Pap test slides. The accessibility provided by digital cytology can improve the quality and efficiency of cytopathology services, primarily by getting the right cytopathologist to remotely look at the right slide. If you can get the right specialist to look at the right slide at the right time, it alleviates the need to ship slides, wait for glass slides, or transport pathologists [3].

This paper will discuss recent advances in the field of digital imaging in cytopathology, its various applications and potential uses in cytopathology, and the current limitations and barriers of digital imaging in cytopathology.

## 2. Whole Slide Imaging

Whole slide imaging (WSI) is a digital imaging modality that uses computerized technology to scan and convert pathology and cytology glass slides into digital images (digital slides) that can be viewed on a computer using viewing software [3, 17, 18]. Viewing the digital images mimics a light microscope, which allows a user to scan from field to field and increase or decrease (zoom in/out) the magnification. Therefore, this is also known as “virtual microscopy”. Virtual microscopy is defined as the simulation of microscopy. A real microscope has four functions: display, pan (move around), zoom (different magnifications), and focus. These are the functions that are now simulated by virtual microscopy using WSI, in which “virtual slides” are displayed, panned, zoomed, and focused using a virtual slide viewer on a computer monitor without the need for a microscope [1, 3]. 

WSI is becoming increasingly robust with advanced capabilities including 3-D technology [19, 20]. The *Z* function offers manual-focusing capability of virtual slides in different planes [21]. This is necessary for certain cytological specimens with thick preparations or cell clustering such as hyperchromatic crowded groups in a Pap Test or cell clusters in fluid cytology or urine cytology [3, 6, 22, 23]. We now have technology that is a capable of scanning up to 100 planes of *z* elements. 

**Figure 2:**
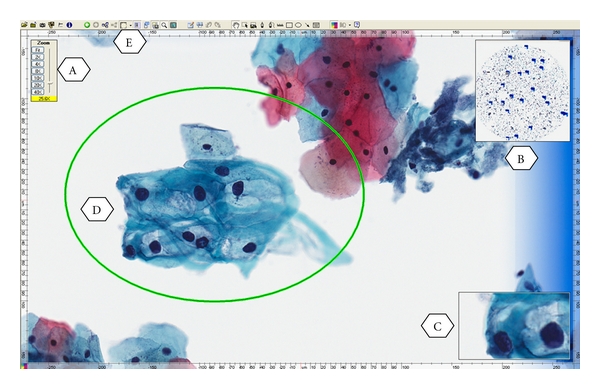
A whole slide image (digitized slide) of LSIL from a ThinPrep Pap Test illustrating the viewer software provided by the vendor to allow for remote viewing and manipulation of images by the cytopathologist. (A) zoom slider, (B) thumbnail image, (C) magnified field, (D) circled area is the annotation layer information used to mark up areas of interest, (E) drawing tool bar.

Current WSI technology provides rapid high-quality image capture and storage, supporting image viewer software. This technology has started to have a significant impact on cytopathology practice in various aspects including rendering a primary diagnosis from a WSI as well as telecytopathology, performing cytology quality assurance, cytology education, and competency assessment of trainees (Figure 2) [6, 7, 24–28]. Numerous recent applications are available with advance capabilities which include the ability to focus up and down the *z*-axis, decrease the time for scanning to only a-few-minute range, and handle large number of slides with a slide feeder of up to 300 slides [6, 16, 20, 27, 29]. Therefore, WSI could provide additional educational benefits to using glass slides. Tables 1 and 2 list advantages and disadvantages of WSI.

## 3. Applications of WSI in Cytology Education and Training

Digital imaging is beginning to replace the traditional classroom with microscopes in medical education including cytopathology [2, 6, 30, 31]. Digital imaging undoubtedly offers significant advantages over the traditional light microscope in education and training. Cytology glass slides are often irreplaceable compared to histological slides, because no recut slides are available (except for cell blocks) as in histological preparations. Also, the colors of stains fade over time, glass slides can be easily broken or lost, the slides can be used only by one person at a time, and a microscope is needed. The main advantage of WSI is that the images are ready anywhere and are easily accessible. A web-based virtual slide library can be permanently stored, and it enables users to review cytological educational material “anytime, anywhere” without microscopes or glass slides. By contrast, the traditional microscopy classroom is costly to set up to maintain, and high-quality cytology glass slides are impossible to duplicate or replace [32]. WSI is sufficient for cytologists to make reliable diagnostic decisions [6, 31, 33, 34]. 

The digital image quality and scan speed to acquire a WSI have greatly improved. Important advantages of virtual microscopy are that all users view the same image, and that images are easy to distribute and share over the internet or as DVDs [35]. WSI technology offers the ability to introduce effective online cytology educational programs and online cytology atlases such as the USCAP Virtual Slide box, which offers unknown cases in anatomic pathology and cytopathology. The International Academy of Cytology (IAC) recently offered several digital educational materials on their web site including cases with virtual slides and static images and online lectures, seminars, and workshop [3, 6].

A potential disadvantage of using WSI is that it initially may take a longer period of time to view cases as compared to glass slides. The speed of loading digital images is dependent upon the speed of the user's network and computer. However, rapid advances in information technology have diminished some of these hurdles. The adoption of digital slides in cytopathology practice will take time and may partially or completely replace glass slides in the future. Even though, in the current time period, pathology and cytology training continues on glass slides, efforts should be made to expose trainees to this new technology. The American Board of Pathology has been using virtual slides for a subset of microscopic questions for a number of years. In addition, virtual microscopy has been widely used in surgical pathology and more recently in cytopathology practice as indicated earlier [3, 6, 22].

**Figure 3:**
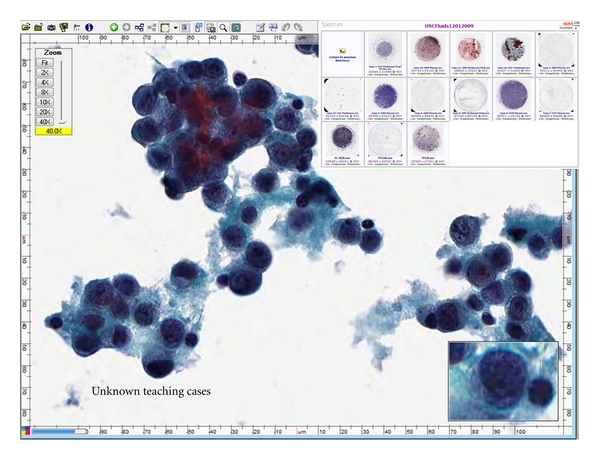
An example of a teaching conference created using whole-slide images. The viewer allows for easy manipulation of images while the user can select from a list of cases that are part of the software. The image of what is a WSI shown with the Aperio ImageScope viewer. Top right shows thumbnail digital images of scanned slides made available via hyeperlinks using an Oracle server. Content related to each scanned slide is incorporated using ColdFusion (Adobe) software.

WSI has been successfully used for teaching cytopathology and surgical pathology and accordingly integrated into academic practice [3, 22, 35]. The use of digital slides for cytology education adds new dimensions to accessibility [35]. Comprehensive digital slide libraries are accessible from home. Slides may be annotated and shared by participants such as pathology residents before conferences [2]. With digital slide conferences, there are many advantages such as improved accessibility to large teaching sets and enhanced annotations with related clinical materials such as radiology images. Due to the increase in usage of WSI in pathology and cytology education, adequate digital pathology and cytology training is becoming a necessary component of pathology resident training. Once the necessary slides in cytology are digitized, an online course or even a “virtual” rotation may be created for residency education [12, 32, 36] (Figure 3). The concept of virtual rotation for cytopathology is similar to some online courses in pathology such as the virtual rotation in pathology informatics [37]. The course includes didactic lectures given by experts in the field as well as online modules and courses. The online course can accommodate various rotation structures as a self-paced rotation and is available to all pathology residency programs. Some residency programs have started to incorporate this virtual rotation in their resident training [37]. The same resource and expertise can be used to add didactic lectures to the digital teaching set program to create a virtual subspecialty rotation in cytopathology [35]. Similar digital teaching sets may be developed in almost all specialties of anatomic pathology such as the virtual slide box from the USCAP.

 The digital teaching sets can be accessed remotely from anywhere with network connections. These slides should be deidentified before scanning and placement on a web site. The step of deidentification is critical to make the whole-slide images available to general users. The digital teaching sets along with other components like pre- and postrotation exams and virtual rotations can provide valuable educational opportunities to pathology residents and trainees nationwide and internationally, which may potentially make standardization of pathology teaching possible in the future [12, 28, 38].

## 4. Applications of WSI in Cytology Quality Assurance (QA)

Quality assurance (QA) is an important part of cytopathology practice and is mandated by the College of American Pathologists (CAP) in their accreditation of laboratories. Although the CAP publishes a series of checklists to guide Cytopathology QA, the specific implementation is intentionally left flexible to accommodate the wide variety of pathology practices that exist. Typical approaches to cytology QA include a review of cases in a variety of settings such as intradepartmental, interdepartmental, or extradepartmental review and second opinion requested by patients or clinicians [10, 39]. Discrepancies should be noted and resolved between pathologists and, if necessary, amended reports or addenda issued. There is good reason behind extensive QA programs in anatomic pathology and cytopathology since Raab et al. [26] have estimated that the actual error rate likely ranges from 1% to 5%, and in a study of self-reported discrepancies among 72 institutions, 6.7% of anatomic pathology diagnoses were found to be discrepant at second review [26]. In 1% of these cases, a significant clinical event occurred as a result of these discrepancies. Statistics based upon second review in the same institution are likely to reflect an underrepresentation of errors because traditional case overreads are associated with a number of potential biases such as the reviewer often knowing the original diagnosis and/or the identity of the sign-out pathologist [22, 24, 26, 27].

 Furthermore, it has been shown that knowledge of the original diagnosis affects the sensitivity of review in cytopathologic specimens. This could hide or even enhance local biases and could be a significant problem, especially for large, multiple-facility health systems that would like to establish a uniform level of quality across their enterprise. A major hindrance to establishing a multifacility QA program is the expense and difficulty of moving and managing slides between facilities, especially if the QA is to be done close to the sign-out date. Automated WSI, in which all the slides in a case are imaged in their entirety at high resolution and made available to cytopathologists on a network, is a modality that may prove useful in cytopathology case review [40]. A digitized case could allow a QA system to hide the original sign-out pathologist and, if desired, the original diagnosis. More importantly, however, digital slides available on a network can mitigate the problems of glass slide logistics and, by so doing, enable routine multifacility cytopathology QA [3, 4, 10, 25, 41, 42].

## 5. Automation in Pap Test Screening

The Pap test has been remarkably successful as a cancer screening tool. Manual screening of large numbers of Pap test slides has several drawbacks including cytotechnologist staffing shortages, ergonomic problems, and the consequences of false negative cases. As a result, technology was employed to automate this screening process. Automated Pap test screening systems have developed under two major system designs: (1) those that perform primary screening without cytotechnologist interaction and (2) an interactive design that serves as the “cytotechnologist's cytotechnologist,” in which both the cytotechnologist and the computer depend upon each other for Pap test interpretation [22, 43–48].

With such interactive screening systems, the cytotechnologist benefits from improved overall job satisfaction, decreased fatigue, and increased throughput. This leads to increased laboratory productivity, provides focused time spent on challenging cases, and directs attention directed to relevant fields of potential abnormality, all resulting ultimately in increased sensitivity. However, with more cases to screen comes the need to address new workload limits. There are several limitations of the digital workflow. These include incorporating the imaging station into the workflow. Also, during the initial installation, calibration, and training, users may be resistant to these systems, especially if the abnormal fields are not the most diagnostic. In addition, there may be specimen adequacy issues and the diagnosis of infections may be limited to field of view. There may also be reimbursement and/or billing issues to be dealt with [22].

Automated screening systems include, as mentioned above, primary screening systems and interactive screening systems. The primary screening system, such as BD FocalPoint Slide Profiler (formerly AutoPap), is a self-contained onsite unit with slides scanned at varying objective levels. The computer processors assign scores for each field of view (FOV). The negative slides receive no human review and are archived and if any abnormalities detected, then the slides require human review [46]. The interactive screening system such as the ThinPrep Imaging System [47] and BD FocalPoint GS [46] scan the entire slide (e.g., 120 fields). The data is then processed using imaging algorithms, coupled with a location-guided workflow process (“pap map”). The cytotechnologist attention is driven using *x*-*y* axis relocation to significant fields (e.g., 22 FOVs). The BD FocalPoint GS Imaging System is designed to process BD SurePath Pap test slides, initially providing slide ranking and adequacy information to cytotechnologists, followed by relocation on a microscope with an automated stage of the 10 microscopic FOVs, having the highest probability of containing an abnormality. An additional FOV is presented initially to allow for location verification/calibration before the FOV screening process is begun. Following review of the FOVs, if no abnormality or adequacy abnormalities or issues are identified, the case may be signed out as negative for intraepithelial lesion or malignancy (NILM), if not subject to quality control rescreening. Slides in which adequacy issues or potential abnormal cells or patterns are identified are subjected to a full manual review [46]. The system selects 15% of the highest ranked NILM slides for QC review. Such a nonrandom rescreening process has been shown to be more effective in identifying false-negative cases than is the current standard CLIA '88-mandated random QC process. Slides, found to be potentially abnormal after primary guided screening, are reviewed by pathologists as per the current standard of practice in gynecological cytology. Recent data from the Manual Assessment Versus Automated Reading In Cytology (MAVARIC) trial suggests that the automation-assisted reading was 8% less sensitive than manual methods for the detection of CIN 2+ methods [49]. Other studies have suggested improved sensitivities over manual methods [13, 43, 46]. Additional studies are needed to fully assess the disadvantages and advantages of automated screening methods using digital imaging.

## 6. Proficiency Testing in Cytology and Digital Imaging

Significant potential exists for the use of digital images in gynecologic proficiency testing, irrespective of whether a test format persists or a continuing education setup is adopted in the future [22]. The current gold standard is manual screening or review of glass slides. Digital imaging is being recommended by several investigators [46]. 

The federal government has mandated a national proficiency testing program for gynecologic cytology as a requirement included in the Clinical Laboratory Improvement Amendments of 1988 (CLIA '88). This proficiency testing program has been implemented. It consists of submitting pathologists and cytotechnologists to periodic tests using well-standardized glass slides or computerized methods. Implementation of this CLIA '88 mandate on a national level requires the availability of a large number of well-vetted test glass slides, complex logistics to handle the administration of the test to thousands of laboratories dispersed in a large geographic area, and management of the data collected from thousands of participants. The College of American Pathologists (CAP) developed the interlaboratory comparison program in gynecologic cytology in 2003 [50]. These reference slides have been previously carefully screened and validated by at least 3 referees who are members of the CAP Cytopathology Resource Committee, and they are field-validated by at least 20 participants before they become acceptable for use in the PAP program. The participants select diagnoses from a coded answer sheet with graded diagnoses that are equivalent to the diagnostic terminology. The CAP has also recently offered online educational material using virtual slides in both GYN cytology (The CAP PT program: Gynecological Cytology PT Program) and in non-gynecological GYN (The NGC Online Activity). These programs also offer the participants choices in viewing static images from the same cases with extensive discussion of the differential diagnoses, illustrated images of the differential diagnoses, and applicable ancillary studies [42, 50].

## 7. Telecytology 

Telecytology, a component of the broader field of telepathology, is the practice of cytology at a distance, by using telecommunication (e.g., the Internet) to transmit digital images [1, 3, 21, 40, 51–56]. Telecytology was made possible by the emergence of digital imaging technology and computers with high processing capacity. There are three modes of telecytology: static (store-and-forward), dynamic (real-time), and hybrid systems [6, 41]. Telecytology systems include digital/video microscopy (cameras attached to microscopes), robotic systems (with a remotely controlled microscope stage), and whole slide (virtual) scanners. The latter two systems, albeit more expensive and demanding on networks, offer the telecytologist remote control of better quality digital images [51]. They provide access to all (and not just selected) areas of interest on a slide [3]. Most publications to date have employed static telecytology and/or video microscopy. Both Pap tests and non-gynecological cases are amenable to telecytology for immediate interpretation and second opinion consultation. Recent improvements in diagnostic concordance (accuracy) are linked to advancing technology, user training, and familiarity with such systems. Globally, remote interpretation of digital images has the potential to provide effective screening and clinical triage to individuals in underserved populations [3].

There are a variety of technologies and approaches available for telecytology applications, ranging from simple transmission of static digital images over phone lines or the Internet, to more complicated real-time transmission of live (streaming) images, and finally to the current state of the art—WSI scanning and transmission [3, 6]. Transmission of static images is relatively simple, requiring only a camera and a network connection. Images can be relatively small (in terms of memory and transmission requirements); however, they suffer from representing only limited portions of the specimen and the potential biases of the image acquirer relative to the image observer, who sees what the sender wants them to see. In “easy” cases, this bias may not be an issue, but in more difficult cases (the type that would be more routinely shared in consultation) this biased partial representation might be very much an issue. In addition, lack of focusing ability and the issue of image manipulation (contrast, brightness, and color) may all be important impediments to a successful outcome. Real-time image transmission involves an image stream, sent immediately upon acquisition, and continually updated as the specimen is reviewed [1, 41, 55]. This type of imaging potentially allows for review of the entire slide, with focusing, and changes in magnification as required. In addition, there can be real-time interaction with the sender during the interchange. There are several systems for real-time image transmission, some of which are controlled at the local site, whereas others can be remotely controlled by the distant observer. Such systems allow for an unbiased review of the slide as the observer can either control the review or at a minimum instruct the local site to “move left, go to high magnification, focus,” and so forth. However, such systems can be cumbersome to use, may require large bandwidth network connections, and therefore can be slow [3]. Particularly in cytology situations where screening or review at high magnification is required, slow image refresh rates and remote command transmission can lead to observer frustration and can even overload networks. Such systems have been successfully deployed for case consultation and for rapid cytology evaluations (adequacy assessments) [18, 51]. 

**Figure 4:**
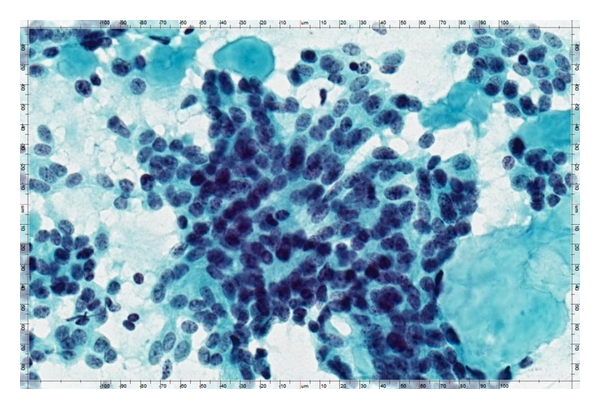
An example of a static image that is acquired from a whole-slide image using the viewer software interface allowing for multiple uses of the acquired image such as for use in telecytology or remote conferences.

WSI technology may offer significant advantages for telecytology applications over static and real-time image transmission. However, these advantages come with some cost. WSI equipment allows for image capture in at least two dimensions with scanning magnification at high enough magnification to produce an image of the entire specimen with similar resolution to what is routinely used in a standard light microscope. Hence, instead of partial images of the specimen, the observer of a WSI can review the entire specimen in a similar fashion to reviewing the actual slide under the microscope (Figure 4). 

WSI files are much larger as compared to a standard static image. The static images might require 3–5 megabytes of memory while WSIs require hundreds of megabytes of memory for a single image. In addition, the 2-dimensional images do not allow for focusing, and this is a particular problem with cytology specimens which are routinely more 3-dimensional than a standard histology tissue section. But, WSI scanners have methods to tackle this problem as well. Multiple scans of the same slide, taken at different focal planes, can be “stacked” into a final composite image (referred to as a “*z*” stack), but as would be expected, each of these planes requires the same amount of memory and as such “*z*” stacks can be slow to load and transmit, leading to significant observer frustration, and storage of significant numbers of such scans requires very large server capacity [3, 22]. 

The technological limitations of WSI for telecytology can be looked at as mere “engineering” problems that may be resolved with technology advancement in the future. In the meantime, 2-dimensional images of cytology specimens can be made to exhibit a more 3-dimensional appearance through multiplane “up-front” scanning followed by a software “trick” that incorporates the best focused image at each pixel into the final 2-dimensional composite (or intercalated) image. Improvements in the focusing of cytology specimens have been significant when using such technology and provide an excellent interim solution to the cytology 3-dimensional problem while the field waits for the technology catche up to a fully focusable image [22, 28, 29]. The major advantage of WSI is that the entire slide is immediately available at all magnifications with a quality close to light microscope analog. The disadvantage is the time necessary for scanning, which can range from 3–5 minutes for a liquid-based slide up to 10 minutes for a conventionally prepared smeared slide and up to an hour(s) for multiplane scanning necessary to generate composite 2-dimensional or “*z*” stacked images [29].

 The images, once they are acquired, may be used for telecytology either to sign out slides or for real-time rapid interpretations, such as adequacy assessments on fine-needle aspiration specimens performed at a remote site not having cytology expertise. Such transmissions could be from office to office within the same campus, but theoretically there would be no difference in the digital environment for these consultations to be from anywhere in the world to anywhere else having a high-speed internet connection. Although imminently possible with today's technology, applications for clinical use will face significant practical challenges to widespread implementation. Information technology (IT) restrictions of institutional firewalls and other security issues, in addition to, routing of HIPAA-protected patient information along with images, will similarly require solutions, and the technology and system barriers required to be overcome in order to make this happen are, at present, formidable.

## 8. WSI for Cytology Slide Archiving

Another clinical use of WSI in cytology is for slide archiving. Institutions receive numerous patient consultations, both for expert opinion and for patient care continuity among institutions, for which slides need to be returned. Receiving institutions can now keep a permanent, near-perfect record of the slides via WSI scanning. In addition, adding slides to digital databases (PACS) will allow their merger with other clinical information, providing a permanent and complete electronic record of all information, including pathologic samples. Telecytology has also been used effectively for distance-based continuing education with teleconferences using static images accompanied by a lecture, real-time microscopy sessions, or tumor boards being broadcast regionally within health care systems or out to other users at large [6, 22].

## 9. Future Applications of Digital Imaging in Cytology and Potential Barriers

In the future, WSI may be adaptable to other uses such as cytology proficiency testing, where having a well-defined, well-validated WSI test would be easier to maintain and distribute than are the current extensive glass slide collections required by PT providers at present. Use of WSI for archiving and presenting rare cases, unusual presentations, and classic examples of entities would be of significant value. Novel telecytology applications already explored involve combinations of automated slide screening with machine acquisition of relevant regions which can be automatically distributed to remote reading stations for review. 

The capability to perform telecytology procedures is currently available, but in a relatively rudimentary stage at present. Problems to be addressed include issue of multiplane (3-D) specimens, scanning speed, bandwidth required for rapid transmission, and IT issues related to firewall security and the transmission of protected patient information. Also, a program needs to be in place to ensure maintenance occurs for these systems. These issues will almost certainly be overcome in the not-too-distant future. In addition, issues of validation are important—we can interpret cytology specimens by these methods as well as by using standard microscopes—the FDA is already considering standards for the practice of telepathology, including methods, equipment, and interpretation. 

Investigators also need to work on the ergonomic issues of image review. Simple mouse-driven computer screens may not be efficient, and other methods of review, such as images in high resolution displayed on large walls or better and bigger monitors at pathology “Cockpits” (where magnification is controlled by the observer moving away or closer), may be necessary to increase efficiency and accuracy—all leading to better acceptance of the method. The day will soon come when all cytologists will have the ability to share images with colleagues easily, efficiently, and quickly. The degree and speed of adoption of new technologies will be variable and will depend on the successful resolution of the many challenges.

## 10. Conclusions

The practice of cytology is evolving rapidly, and cytologists must prepare for tomorrow. In the coming years, several changes such as the advancement of personalized medicine and the emergence of technological advances like digital pathology will greatly impact how a cytologist performs his/her job. Traditional microscopy may eventually become obsolete. Glass slides may be replaced by high-definition digital images that can be viewed using a computer display screen. we are closer to this stage not only through already existing WSI technology, but also through efforts of many institutes and vendors who continue to build newer, faster, and cheaper scanners and sophisticated software to improve digital pathology workflow.

With the potential of having all cytology slides, cell block slides, and ancillary studies scanned to produce WSI, cytologists and cytopathologists will see a significant impact on their practices in cytopathology. It will allow them to access, review, share, and analyze digital data with computer assisted algorithms and sign out their cases online, anywhere and anytime with computer access. It will allow them to perform cytology QA and proficiency testing and participate in educational programs more easily. Cytotechnologists and cytopathologists may have the option of remote accessibility of materials and telecytology. QA cases may be done by a remote cytology laboratory without the influence of knowing the original diagnosis or factors that may create a bias in their diagnostic decision. There may be applications that provide intelligent content-based image retrieval methods, in which informatics and IT infrastructure may help to find other cases with similar cytomorphological appearance. Advances in WSI may utilize digital image processing techniques to reveal details that are not easily available by looking at the glass microscope slide. The cytopathologist will use digital imaging technologies of the future to function as a primary diagnostic consultant to the patient by integrating multiple sources of information such as molecular pathology and flow cytometry and correlating this with cytopathology.

## Figures and Tables

**Table 1:** Advantages of whole slide imaging in cytopathology practice.

(1) Primary diagnosis (telecytology)	
(2) Remote second opinion consultation	
(3) Educational activity within the institution or remotely, for example, CAP online program	
(4) Archiving interesting and legal cases (digital cytology slides replication)	
(5) Quality assurance	
(6) Educational conferences such as tumor boards (locally or remotely)	
(7) Online cytology proficiency testing	
(8) Online board exam or certification	
(9) Detailed image analysis and cytomorphometry	
(10) Annotation of various entities on the slides for teaching purpose	
(11) Easy acquisition of static images from whole-slide images	
(12) Provide cytopathology services to remote hospitals	
(13) Gains access to cytology subspecialty expertise	
(14) Remote on-site evaluation and triage	
(15) Synchronous consultation	

**Table 2:** Disadvantages of whole slide imaging in cytopathology practice.

(1) Costly: an expensive initial setup and storages	
(2) Limited focusing functions at present	
(3) Scanning time	
(4) Storage: large file size	
(5) Training requirements	
(6) Limited validation studies	
(7) Lack of standardization: multiple vendors, software, and lack of interoperability	
(8) Information technology infrastructure support (bandwidth limitation of networks)	
(9) Professional reluctance to adopt	
